# Effect of Cu Modified Textile Structures on Antibacterial and Antiviral Protection

**DOI:** 10.3390/ma15176164

**Published:** 2022-09-05

**Authors:** Małgorzata Cieślak, Dorota Kowalczyk, Małgorzata Krzyżowska, Martyna Janicka, Ewa Witczak, Irena Kamińska

**Affiliations:** 1 Department of Chemical Textile Technologies, Lukasiewicz Research Network-Lodz Institute of Technology, Maria Sklodowska-Curie 19/27, 90-570 Lodz, Poland; 2 Department of Nanobiology and Biomaterials, Military Institute of Hygiene and Epidemiology, Kozielska 4, 01-163 Warsaw, Poland

**Keywords:** textile structures, polyester fabric, cotton fabric, copper, magnetron sputtering system, antibacterial activity, antiviral activity, comfort properties, fabric modification

## Abstract

Textile structures with various bioactive and functional properties are used in many areas of medicine, special clothing, interior textiles, technical goods, etc. We investigated the effect of two different textile woven structures made of 90% polyester with 10% polyamide (PET) and 100% cotton (CO) modified by magnetron sputtering with copper (Cu) on bioactive properties against Gram-positive and Gram-negative bacteria and four viruses and also on the some comfort parameters. PET/Cu and CO/Cu fabrics have strong antibacterial activity against *Staphylococcus aureus* and *Klebsiella pneumonia*. CO/Cu fabric has good antiviral activity in relation to vaccinia virus (VACV), herpes simplex virus type 1 (HSV-1) and influenza A virus H1N1 (IFV), while its antiviral activity against mouse coronavirus (MHV) is weak. PET/Cu fabric showed weak antiviral activity against HSV-1 and MHV. Both modified fabrics showed no significant toxicity in comparison to the control medium and pristine fabrics. After Cu sputtering, fabric surfaces became hydrophobic and the value of the surface free energy was over four times lower than for pristine fabrics. The modification improved thermal conductivity and thermal diffusivity, facilitated water vapour transport, and air permeability did not decrease.

## 1. Introduction

Textile structures with various bioactive properties are used in many areas of medicine, special clothing, interior textiles, technical goods, etc. [1,2,3,4,5]. The research interest in this aspect has dynamically increased during the COVID-19 pandemic. New solutions in the field of textile engineering, modifiers and bioactive functionalization methods aim to reduce the risk of SARS-CoV-2 infection by using bio-barrier materials for various purposes (masks, special clothing, surface coverings, tents, etc.). One of the methods of functionalization is the use of particles, compounds and complexes, including different forms of copper [6,7,8]. Copper based modifiers have a well-documented mechanism of antimicrobial activity against bacteria, fungi and viruses [9,10,11,12,13,14,15,16,17,18,19,20,21]. Most of the research is focused on the assessment of the bioactive properties of both copper structures and copper metallized materials by various techniques against selected bacteria or viruses [22,23,24,25,26,27,28,29,30,31,32].

The mechanism of antimicrobial activity of copper is complex and shaped by many factors, but generally it is claimed that, when bacteria or viruses are on the copper surface, Cu ions damage the cell membrane or viral envelope. The destroying of microbes is accelerated by the forming of free radicals [33,34]. Warnes and co-authors state that copper is more effective in antimicrobial activity than other used metals, due to it having a free electron in its outer orbital shell of electrons that easily takes part in oxidation-reduction reactions [33,34]. Therefore, in the development of materials protecting against viruses, the interface interaction solid surface-protein is an important issue. In the case of viruses the inactivation process is more effective on hydrophobic surfaces with higher roughness, which is connected with the higher affinity of protein to adsorption on such surfaces [35,36,37,38]. Bioactive textiles protecting against microorganisms should also ensure comfort of use. Achieving full comfort and maintaining a bio-barrier is difficult, but proper design of the textile structure can significantly improve some comfort parameters.

The aim of the work is to investigate the effect of two different textile structures modified by magnetron sputtering with copper on bioactive properties against two bacteria strains and four viruses and on the comfort parameters. The presented approach is new and may have significant value in the development of textile structures as bioactive materials.

## 2. Materials and Methods

Two fabrics with different structure and raw material composition were used in the study. The characteristics of the fabrics are presented in Table 1.

### 2.1. Modification of Fabrics

**Figure 1:**
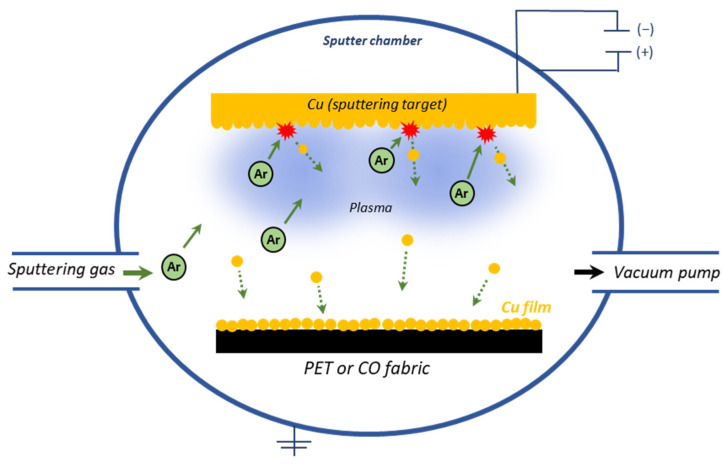
Schematic diagram of DC magnetron sputtering system.

One-side modification of fabrics with copper was carried out using the DC magnetron sputtering system, (P.P.H. Jolex s. c., Czestochowa, Poland) which enables continuous (“roll to roll”) or stationary application of thin coatings of metals, alloys and oxides in single layers or multilayer systems. The magnetron sputtering system is equipped with a pulse current source with a power of min. 12 kW and a maximum voltage of 1.2 kV with an adjustable group frequency from 50 Hz to 5 kHz. The process was carried out in an inert gas—argon using the copper target with a purity of 99.99% (Testbourne Ltd., Basingstoke, UK) in the conditions presented in Table 2. A schematic diagram of the applied DC magnetron sputtering system is shown in Figure 1.

### 2.2. Microscopic Analysis

SEM microscopic analyses were carried out on a scanning electron microscope VEGA3 (TESCAN, Brno, Czech Republic) with magnification of 100×, 2000×, 5000× and 10,000×, using the high vacuum mode, secondary electron (SE) detector and the energy of probe beam of 20 keV. The samples were sputtered with gold on the Quorum Technologies Ltd. vacuum device (Laughton, UK). The elemental analysis was performed on the EDS INCA Energy spectrometer (Oxford Instruments, High Wycombe, UK) on the samples without sputtering with gold. X-ray microanalysis was done under air pressure of 10 Pa, using an accelerating voltage of 20 kV, backscattered electron beam (BSE) and the SmartMap function. For each sample, maps of the distribution of elements, the sum spectrum as well as weight and atomic percentages of elements, were prepared. The maps were determined for the elements C, O, Ti and Cu for the Kα line with the excitation energy E = 0.28 keV, E = 0.52 keV, E = 4.51 keV and E = 8.04 keV, respectively.

The microscopic analysis of the topography was performed on an OLYMPUS DSX1000 digital optical microscope (Tokyo, Japan). The samples were tested in light and dark field (MIX) with the possibility of adjusting the intensity and direction of incidence of light and with the use of the simple polarization (PO) method. 3D acquisition was used for all images by registering successive images while moving in the direction of the Z axis. The tests were performed at 100× magnification. Based on the 3D topography of the fabric surface, the following parameters of its roughness were determined: Sq—Root mean square height, Sa—Arithmetical mean height, Sp—Maximum peak height (peak), Sv—Maximum pit depth (valley) and Sz—Maximum height (Sp + Sv).

### 2.3. Determination of Cu Content

In order to determine the copper content, the fabric sample was mineralized in a microwave mineralizer (Magnum II, Ertec, Poland). The mineralized sample was then dispersed in purified water. The content of copper in the dispersion was determined by means of an atomic absorption spectrometer with flame atomization (SpectrAA 250 Plus, Varian, Australia).

### 2.4. Antibacterial Test

The bacteria strains of Gram-positive *Staphylococcus aureus* (ATCC 6538) and Gram-negative *Klebsiella pneumoniae* (ATCC 4352) were used in the study. The test was performed according to PN-EN ISO 20743:2013-10 Determination of antimicrobial activity of finished products with antibacterial finish. The pristine textile structures were used as reference materials. Assessment of antibacterial activity (A) was carried out according to EN ISO 20743:2013, where 2 ≤ A < 3 means significant and A ≥ 3 means strong bioactivity.

### 2.5. Antiviral and Cytotoxicity Tests

The following pathogens were tested: herpes simplex virus type 1 (HSV-1) McKrae strain (Gothenburg University, Sweden), vaccinia virus (VACV) WR strain (ATCC VR-1736), influenza A virus H1N1 (IFV) (ATCC VR-1736) and mouse coronavirus (MHV) JHV strain (ATCC VR-765). Tests with HSV-1 and VACV were performed in Vero cells (ATCC CCL-81) cultured in Dulbecco’s Modified Eagle’s (DMEM) Medium with GlutaMAX supplemented with 10% fetal bovine serum (FBS), 100 units/mL penicillin, 100 µg/mL streptomycin (Thermo Fisher Scientific, Waltham, MA, USA). Vero cells were inoculated with cryopreserved HSV-1 or VACV. The virus infection was monitored by observable cytopathic effects (CPE). The virus stock infectivity titer (plaque forming unit (PFU)/mL) was determined in Vero cells inoculated with serial dilutions of virus suspensions. After 48 h, infected cell cultures were stained with 1% crystal violet and used to determine the number of cytopathic effects per mL (PFU/mL) [39]. Tests with IFV were performed in MDCK (NBL-2) (ATCC^®^, Manassas, VA, USA, CCL-34) cells cultured in DMEM medium with antibiotics and 10% FBS (Thermo Fisher Scientific). MDCK cells were inoculated with cryopreserved IFV, then cultured for 72 h and monitored for observable cytotoxic effects. Tests with MHV virus were performed in NCTC clone 1469 cells (ATCC^®^ CCL-9.1), cultured in Minimum Essential Media (MEM) with antibiotics and 10% FBS (Thermo Fisher Scientific). The infected cultures were observed for 24 h for the presence of CPE. For MHV and IFV, TCID50/mL (Spearman-Kärber method) [40] was used to determine the concentration of the inoculated virus based on the outcome of the end-point dilution resulting in the CPE of the MDCK (IFV) or NCTC (MHV) cells cultured in 96 well plates. The mouse beta-coronavirus (MHV) was chosen in context of future research concerning the SARS-CoV-2. It is an enveloped virus with a positive-sense RNA genome in the *Coronaviridae* family and has been studied widely as a model of viral pathogenesis [41].

The study was performed according to the standard 18184:2019 Textiles—Determination of antiviral activity of textile products. As a negative untreated test control material, the same fabrics but without modification were used, hereafter referred to as control fabric. The control and test samples were cut into 1.0 g pieces and autoclaved to sterilize them. The samples were then inoculated with 0.2 mL of the viral inoculum. The control samples were subjected to washout immediately after inoculation, while experimental samples were incubated at room temperature (RT) for 2 h. Each control and test sample was prepared in triplicates for each time point. All samples were washed with 20 mL of the neutralizing solution (complete cold medium), followed by 1 min of vortexing. Aliquots of the neutralizing solutions were next used to determine the infectious titer of the recovered virus by PFU/mL (HSV-1, VACV) or TCID50/mL (IFV, MHV) method. 

The antiviral activity (Mv) value was calculated as follows: Mv = −log(Vc/Vt) = −[log(Vc) − log(Vt)], where log(Vc) = the common logarithm average of three infectivity titer values immediately after inoculation of the control sample; log(Vt) = the common logarithm average of three infectivity titer values immediately after the 2 h contact time with the tested samples. According to the standard 18184:2019, good antiviral activity is described as 3.0 > Mv ≥ 2.0.

In order to control for potential cytotoxicity caused by eluting molecules from the test samples during vortexing to the neutralizing solution, the following test was conducted: 20 mL of the neutralizing solution was added to non-virus inoculated control and test samples, incubated for 2 h and after vortexing, aliquots from these solutions were added to the Vero cells. Cytotoxicity was then monitored with MTT cell viability assay according to the producer’s manual (Thermo Fisher Scientific) (where MTT is (3-(4,5-dimethylthiazol-2-yl)-2,5-diphenyltetrazolium bromide). 

### 2.6. Determination of Wettability and Surface Free Energy

The contact angle θ was determined using a PGX goniometer (Fibro System AB, Sweden). Three wetting liquids with known values of surface free energy (γ_L_) and its components (γ_L_^d^,γ_L_^p^) were used: water, W (γ_L_ = 72.80 mJ/m^2^, γ_L_^d^ = 21.80 mJ/m^2^, γ_L_^p^ = 51.00 mJ/m^2^), formamide, F (γ_L_ = 58.00 mJ/m^2^, γ_L_^d^ = 39.00 mJ/m^2^, γ_L_^p^ = 19.00 mJ/m^2^), hexane, H (γ_L_ = 18.40 mJ/m^2^, γ_L_^d^ = 18.40 mJ/m^2^, γ_L_^p^ = 0 mJ/m^2^). The droplet volume was 3 µL, temperature 22.3 ± 1 °C, relative humidity (RH) 40 ± 1%. Three repetitions for each sample were made and mean values and standard deviation were determined. Based on the mean value of the contact angles, the surface free energy and its dispersion and polar components were calculated according to the Owens-Wendt method [42].

### 2.7. Air Permeability Measurement

Air permeability was measured in accordance with the standard PN-EN ISO 9237:1998, Textiles. Determination of permeability of fabrics to air, using pressure difference of 100 Pa, temperature of 21 °C and RH = 64%.

### 2.8. Water Vapour Permeability Measurement

Water vapour permeability (WVP) was measured in accordance with the standard PN-EN ISO 11092:2014-11, Textiles. Physiological effects. Measurement of thermal and water-vapour resistance under steady-state conditions (sweating guarded-hotplate test).

### 2.9. Testing of Comfort Parameter

Two comfort parameters, thermal conductivity, λ (Wm^−1^K^−1^) and thermal diffusivity, a (m^2^s^−1^) were determined using the Alambeta device (Sensora, Praha, Czech Republic) [43]. The test consists of measuring the amount of heat flowing through the sample, placed with a constant pressure of 200 Pa ± 10%, between two plates—the upper one, heated to a temperature of 32 °C, and the lower one with a temperature of 22 °C. For each sample, five repetitions of the measurement were made and mean values and standard deviation were determined. The samples were tested with the Cu modified side in contact with the heated plate.

## 3. Results and Discussion

### 3.1. Analysis of Textile Structures and Cu Sputtering Effect

**Figure 2:**
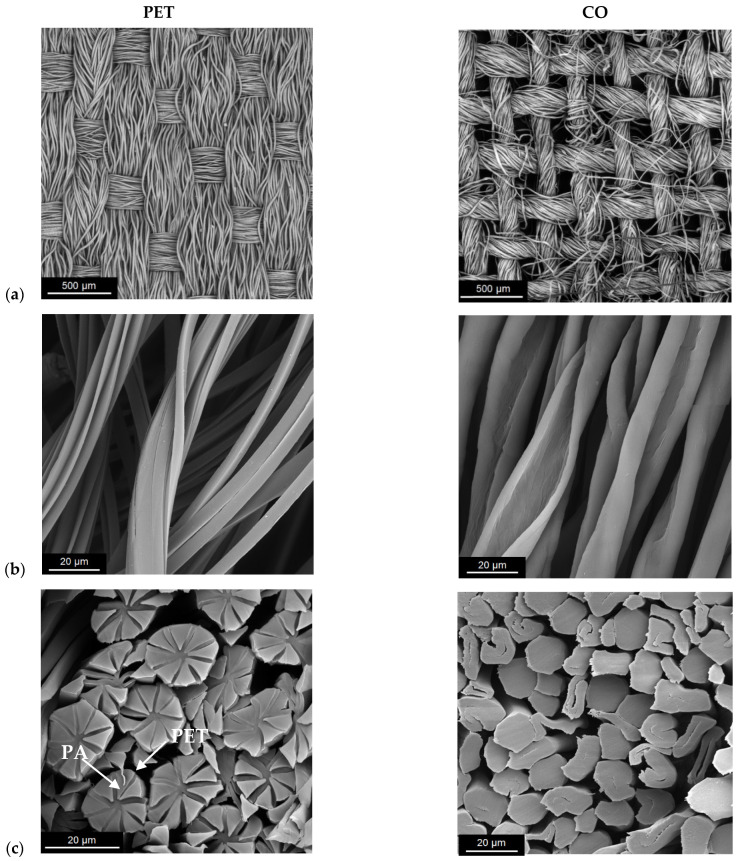
The SEM images of textile materials used in the study: (**a**) woven fabrics, (**b**) longitudinal and (**c**) cross section views of the fibers.

The images of the pristine fabrics presented in Figure 2 show differences in the structures of fibers and fabrics.

**Figure 3:**
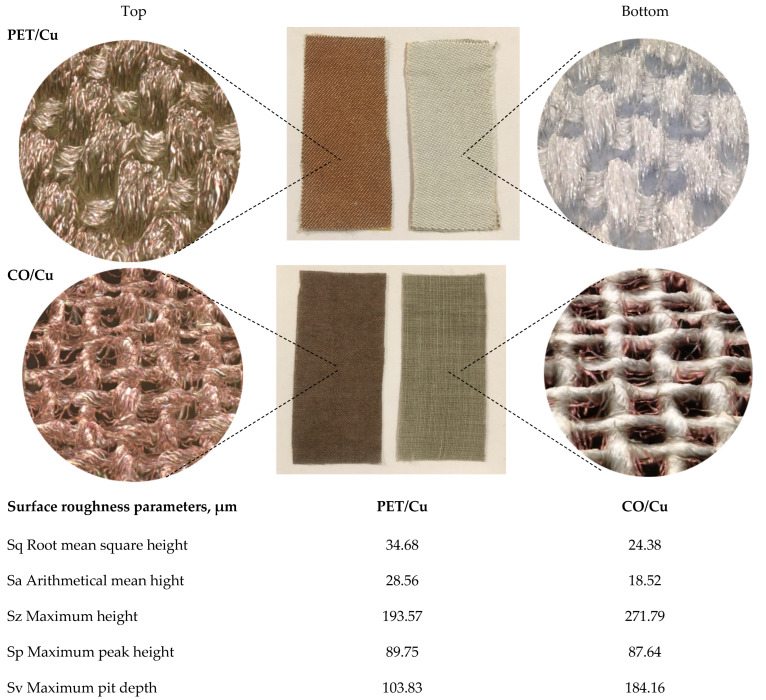
Photos and the microscopic 3D images (magnification × 100) and surface roughness parameters of the Cu modified fabrics.

**Figure 4:**
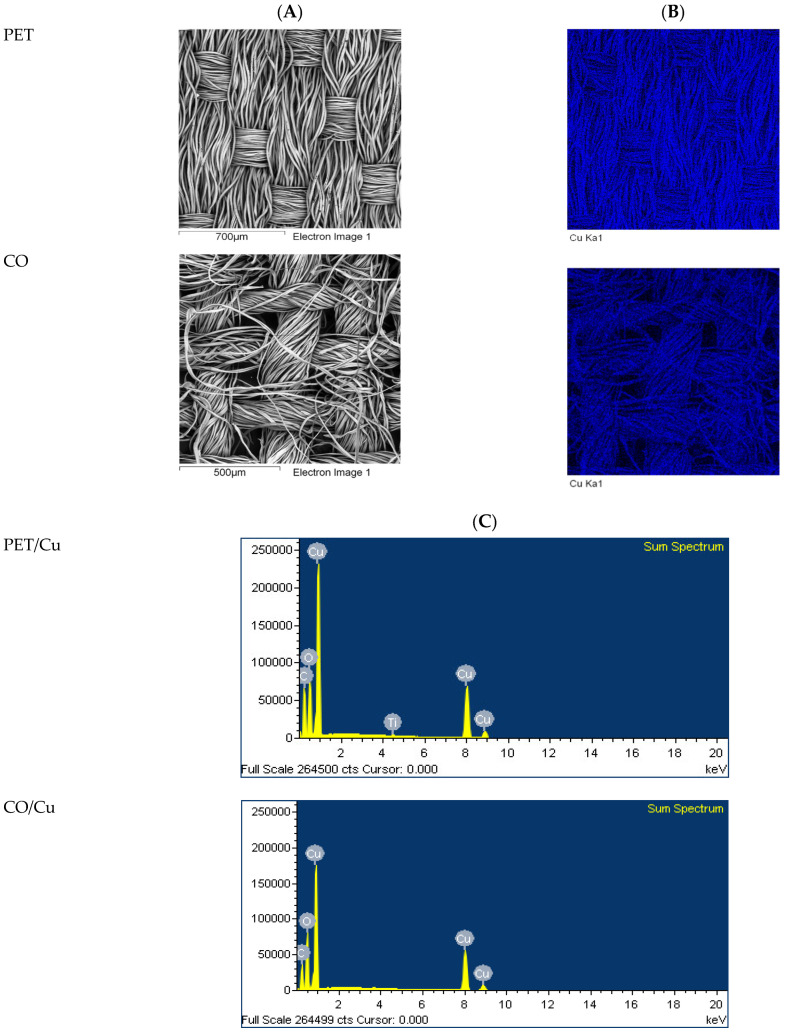
Results of SEM/EDS analysis: SEM images (**A**), exemplary maps of Cu distribution (blue) (**B**) and sum spectra (**C**).

Results of microscopic analysis confirm the complete coverage of the top fabric surface with Cu (Figure 3 and Figure 4). Their topography is different, but well developed, with high roughness. On the bottom surface of the PET/Cu fabric there is no copper. In the case of the bottom surface of CO/Cu fabric, protruding fibers of the weft and warp yarns covered by Cu are visible. The mean values of Cu content determined by the ASA method are 15.2 and 12.6 g/kg for PET/Cu and for the CO/Cu fabrics, respectively. The percentages of Cu, determined by the SEM/EDS analysis (Figure 4), are 38.9 wt% and 43.4 wt%, for PET/Cu and for the CO/Cu fabrics, respectively (Table 3). The presence of Ti in PET fabric is related to the production process of bicomponent yarn. The differences in the Cu content depend on the fabric’s structure. Under the same conditions of modification (Table 2), more copper particles were deposited on the dense PET fabric surface than on the CO fabric surface. In the case of cotton fabric, some of the Cu particles passed through the spaces between the weft and the warp.

### 3.2. Antiviral Activity and Toxicity

The tested fabrics showed different antiviral activity (Table 4). According to the standard 18184:2019, Textiles—Determination of antiviral activity of textile products, good antiviral activity is described as 3.0 > Mv ≥ 2.0. CO/Cu fabric showed good antiviral activity in relation to VACV, HSV-1 and IFV, while its antiviral activity against MHV was weak. PET/Cu fabric showed weak antiviral activity against HSV-1 and MHV. 

**Figure 5:**
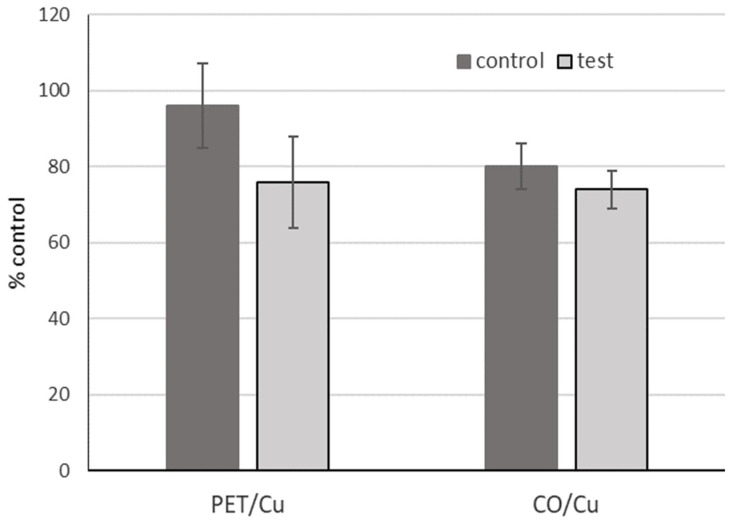
Toxicity of tested samples with MTT method (Cell viability versus control).

Both PET/Cu and CO/Cu modified fabrics showed no significant toxicity (Figure 5) in comparison to the control medium and pristine samples (*p* ≥ 0.05).

### 3.3. Antibacterial Activity

Both modified fabrics are characterized by strong antibacterial activity (A) (Table 5), because according to standardized evaluation criteria A ≥ 3. Stronger activity is observed in the case of Gram-negative *Klebsiella pneumoniae*, which may result from differences in the structure and composition of the cell envelope between the Gram-negative and Gram-positive bacteria. Gram-negative bacteria have a thin peptidoglycan layer and an outer lipid membrane. In Gram-positive bacteria the peptidoglycan layer is significantly thicker than in Gram-negative bacteria and an outer lipid membrane is not present [44]. A study by Vaidya and co-workers also showed that copper has stronger activity against two Gram-negative bacteria *Klebsiella pneumoniae* and *Acinetobacter baumanni* than against Gram-positive *Enterococcus faecium* [45]. The minimal bactericidal concentration values for cooper ion solution against the above mentioned bacteria were 15.62, 15.62 and 125.00 mgL^−1^, respectively [45]. Souli and co-workers investigated the antibacterial activity of copper against potent multidrug-resistant Gram-negative pathogens *Escherichia coli*, *Enterobacter* spp., *Klebsiella pneumoniae*, *Pseudomonas aeruginosa* and *Acinetobacter baumannii* and showed a bactericidal effect of Cu 99% for all tested strains [46]. We are aware of differences in methodological approaches used in the above-mentioned studies, but their results clearly indicate the antibacterial activity of copper.

### 3.4. Surface Properties

After modification, the values of the contact angle (Table 6) significantly changed for both fabrics, and their surfaces became hydrophobic. Research into understanding the wettability phenomenon has been going on for many years and new models or improvements to existing ones are proposed. The apparent contact angle is defined as the angle between the apparent solid surface and the tangent to the liquid-fluid interface. For two-dimensional systems there exists a correspondence between this theory and measured contact angles. Wenzel was the first to discuss the influence of roughness on apparent contact angle [47]. However, a more complex model was needed for heterogeneous systems, which was proposed by Cassie and Baxter (the Cassie–Baxter equation) [48].

In the case of three-dimensional systems with no special symmetry, the apparent contact angle may vary from point to point [49].

Marmur states that equilibrium wetting on rough surfaces is discussed in terms of the “competition” between complete liquid penetration into the roughness grooves and the entrapment of air bubbles inside the grooves underneath the liquid [50]. Marmur added a new condition that is necessary for the existence of the heterogeneous wetting model. It is demonstrated that when this condition is violated, the homogeneous wetting regime is in effect, even though the Cassie-Baxter equation may be satisfied.

Li et al. deposited a copper thin film with different microstructures on Si (100) and quartz substrates using a magnetron sputtering system and copper target (99.99% purity) [51]. Based on the measurement of the contact angle with water (5 μL water droplets), they found that developed surfaces exhibit different wettability (water contact angle from 101.3 to 160.5 deg), depending on the sputtering powers and, consequently, the geometry of surface microstructures. They suggested that air can be easily trapped by the microstructures on the surface, and then water cannot intrude into the interspaces among the microstructures according to the Cassie-Baxter model.

Our textile structures are more complex and varied, both in the physical and chemical aspect, than substrates used by Li et al. Nonetheless, the values of water contact angle for PET/Cu and CO/Cu fabrics are 130.2 and 135.0 deg, respectively, and both surfaces are hydrophobic.

The value of the surface free energy and its components determined on the basis of the contact angle values are more than 4 times lower for PET/Cu and CO/Cu fabrics than for pristine fabrics. These changes are mainly related to the decrease in the polar components values by 53.77 and 53.34 mJ/m^2^ for PET/Cu and CO/Cu fabrics, respectively. The values of dispersive components did not decrease significantly, only by 1.83 and 3.02 mJ/m^2^ for PET/Cu and CO/Cu fabrics, respectively.

The change in the surface properties of the modified fabrics can affect an inactivation of tested pathogens. Protein adsorption on a solid surface is a complex process [52]. Antiviral activity mechanism of materials includes inter alia protein-solid surface interactions, which depend on many factors. The adhesion of proteins to different surfaces is determined by physicochemical properties, such as morphology, topography (roughness, arrangement), tension, polarity, charge, electrostatic interactions, pH, specific chemical interactions, hydrophobic interactions, intermolecular protein-protein interactions, temperature, and humidity. Generally, proteins adhere more strongly to a non-polar surface. Anand and co-authors postulate that non-polar surfaces destabilize proteins and thereby facilitate conformational reorientations leading to strong inter protein and protein-surface interactions [37]. This explains the experimental finding that in most cases the affinity of proteins to surfaces increases on hydrophobic substrates and decreases on hydrophilic ones [37,53]. The results of the antiviral effect depend also on the type/structure of pathogen [54]. The herpes simplex virus type 1 (HSV-1) is a member of the human *Herpesviridae* family. The structure of herpes viruses consists of a relatively large, double-stranded, linear DNA genome encased within an icosahedral protein cage (capsid) which is wrapped in a lipid bilayer envelope. The envelope is joined to the capsid by means of a tegument [55]. The vaccinia virus (VACV) is the member of the family Poxviridae. VACV is closely related to other orthopoxviruses such as variola virus and cowpox virus [56]. The influenza A virus H1N1 (IFV) is a member of the family Orthomyxovirus (a group of RNA viruses). This is an enveloped virus with the characteristic spike-like viral glycoproteins—Haemagglutinin and Neuraminidase [57]. The mouse hepatitis virus (MHV), an enveloped virus with a positive-sense RNA genome in the *Coronaviridae* family, has been studied widely as a model of viral pathogenesis [58]. This virus was selected as a model before future testing the developed materials using SARS-CoV-2. SARS-CoV-2 is an envelope virus with a single linear positive-stranded RNA (+ssRNA) genome. SARS-CoV-2 has four structural proteins: the S (spike), E (envelope), M (membrane), which together create the viral envelope, and the N (nucleocapsid) protein, holding the RNA genome [59]. SARS-CoV-2 virions of 50–200 nm in diameter are like spherical nanoparticles with the glycoprotein spikes on their surface, which are responsible for the contact with the solid surface [41].

Copper has shown the ability to destroy viruses, such as influenza viruses, vaccinia virus, noroviruses or human immunodeficiency virus (HIV) [30,60]. Viruses are more susceptible than fungi and bacteria to copper by the lack of repair mechanisms [18]. The viral genome can be directly targeted by copper, especially gene(s) encoding viral protein(s) essential for viral infectivity [18]. Furthermore, ROS (reactive oxygen species) formation contributes to the death of the cell via interaction with viral envelope or capsid [21,30]. Viruses are susceptible to the Cu-induced damage as they do not have the repair mechanisms characteristic for bacteria [15]. Material containing copper has not been tested for antiviral activity against SARS-CoV-2 virus. However, Favatela and co-workers tested antiviral cotton fabrics impregnated with different formulations based on chitosan, citric acid, and copper against HSV-1 and bovine betacoronavirus (BCoV), and found good antiviral activity against HSV-1 and BCoV [61].

### 3.5. Comfort Properties

**Figure 6:**
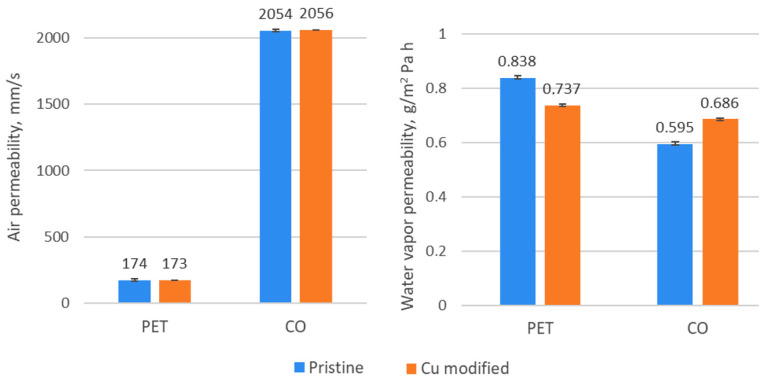
The values of air and water vapour permeability for pristine and Cu modified textile structures.

Materials intended for personal protection, such as masks or overalls, should provide the user acceptable comfort related to the transport of heat and air and water vapour permeability (WVP). The air permeability of fabrics before and after Cu modification was at a similar level (Figure 6). The transport of moisture depends on many factors, i.e., the type and characteristics of the fiber, thickness, surface topography, porosity, type of chemical finish, etc. To design and manufacture the PET fabric a special yarn was used which consists of textured bicomponent supermicrofibers with segmented-pie cross section (80% PET and 20% PA, 16 segments) modified with Ti compounds (Table 1 and Table 3). Pristine PET fabric with dense structure and air permeability of 174 mm/s is characterized by 29% higher value of WVP than pristine CO fabric with loose structure and air permeability of 2054 mm/s (Table 1, Figure 2 and Figure 6). Sampath et al. investigated the effect of filament fineness on comfort properties and found that moisture vapour transmission was higher for fabrics made of finer filaments [62].

Changes in water vapour permeability after Cu deposition, a decrease for PET/Cu and an increase for CO/Cu, are the results of the changes in the structure and physicochemical surface properties of fabrics and susceptibility of the fibers to water sorption. Normal moisture content (at 20 °C and 65% RH) for PET and CO is of 0.3–0.4% and 7.4–9.5%, respectively [63,64,65]. The Cu sputtered surfaces of both fabrics became hydrophobic (Table 6), and their surface free energy value significantly decreased due to the drastic lowering of the polar component values, which favors the transport of water vapour. However, in the case of PET/Cu fabric, the WVP value decreased by 12% as result of the complete covering of the fibers’ surface and filling the interfibrillar space and the space between weft and warp yarns with Cu particles. Pristine cotton fabric has higher susceptibility to water sorption due to the high hygroscopic properties of cellulose (water retention 45–55%) and the fiber structure, characterized by the presence of numerous free spaces, micro-capillaries, micro-pores and diversified lumens [66]. After modification, the WVP value for CO/Cu fabric increased by 15% because a part of the cotton fibers were coated with Cu which reduced sorption, and large spaces between yarns remained unfilled and permeable for water (Figure 3).

Results of the Alambeta test show changes in thermal properties of both fabrics after modification (Table 7). The thermal conductivity of air is 0.025 Wm^−1^K^−1^, and the thermal diffusivity is 0.19 × 10^−4^ m^2^s^−1^, while for Cu 400 Wm^−1^K^−1^ and 1.17 10^−4^ m^2^/s, respectively. After modification, the value of thermal conductivity increased by about 7% for PET/Cu and 2.6% for CO/Cu, and the value of thermal diffusivity by 40.2% and 2.5% for PET/Cu and for CO/Cu, respectively. Thermal diffusivity is a specific property of a material that indicates the rate of temperature changes from one surface to another and assesses how quickly the material reacts to temperature changes. The structure of the PET fabric is compact, which is confirmed by the results of microscopic analyses (Figure 2) and the air permeability (Figure 6). The volumetric porosity for pristine PET and CO fabric is 77.1% and 87.3%, respectively (Table 1). A greater volume of air in the structure reduces the rate of temperature changes. Cu particles improved thermal conductivity and thermal diffusivity by covering the surface of the fibers and filling small spaces between the fibers. In the CO fabric, Cu particles penetrated into the structure between the fibers, but the large spaces between the weft and warp yarns remained filled with air, therefore the increase in the value of both thermal parameters is smaller in comparison with PET fabric.

## 4. Summary

We investigated the effect of two different textile woven structures made of 90% polyester with the 10% polyamide in segmented-pie yarn (PET) and 100% cotton (CO) modified by magnetron sputtering with copper, on bioactive properties against two bacteria strains and four viruses and also on some comfort parameters. Modification of PET and CO woven fabrics with Cu has given them antibacterial properties against *Staphylococcus aureus* and *Klebsiella pneumonia*. The modified fabrics showed different antiviral activity. CO/Cu fabric has good antiviral activity in relation to VACV, HSV-1 and IFV, while its antiviral activity against MHV was weak. PET/Cu fabric showed weak antiviral activity against HSV-1 and MHV. The PET/Cu and CO/Cu fabrics showed no significant toxicity. The values of the contact angle for both fabrics changed significantly after sputtering with Cu and their surfaces became hydrophobic. The value of the surface free energy is over four times lower for the modified fabrics. These changes are mainly related to the lowering of the polar components.

The fabric structure and surface properties play an important role in shaping the comfort properties. Cu modification improved the thermal conductivity and thermal diffusivity of both fabrics. The hydrophobic properties facilitate the transport of water vapour. The applied Cu sputtering conditions allowed for the modification of fabrics without deteriorating their air permeability. The results of the presented research constitute the basis for the design of new textile structures and the conditions for their modification in terms of use as protection of the respiratory system and special coverings. The results of our research and the analysis of the current state of knowledge [67] indicate that raw materials and textile structures and the possibility of their functionalization have a high potential that can be used to design bioactive protective materials with an acceptable comfort of use, dedicated to a specific purpose.

## Figures and Tables

**Table 1:** Characteristics of textile materials.

		Polyester Fabric (PET)	Cotton Fabric (CO)
Raw material		polyester (PET) 90% with polyamide (PA) 10%	cotton 100%
Yarn	warp	PET DTY dtex 110 f 144 (textured microfiber), linear mass of filament 0.76 dtex	tex 8
	weft	PET dtex 167 f (75 × 8) (textured supermicrofiber, biocomponent 80% PET and 20% PA as a spacer in segmented-pie (orange cross section) filament), linear mass of filament 0.28 dtex	tex 8
Wave		¼ (2)—five-thread satin	plain
Treads number/10 cm	warp	469	310
	weft	332	300
Mass per unit area, g/m^2^		130 ± 1	55 ± 1
Thickness, mm		0.41 ± 0.10	0.27 ± 0.10
Volume porosity *, %		77.1	87.3

* The percentage of air in the volume of the fabric.

**Table 2:** Conditions of modification with Cu by magnetron sputtering.

	Pressure, Mbar	Effective Power, kWh	Circulating Power, kWh	Argon Content, %	Number of Passes	Speed, mm/s
PET	2.0 × 10^−3^	2.0–2.2	0.8–1.0	3	15	15
CO	2.0 × 10^−3^	2.0–2.2	0.8–1.0	3	15	15

**Table 3:** Results of SEM/EDS analysis—weight percentage of elements.

Elements, wt%	PET		CO	
	Pristine	Cu	Pristine	Cu
C	63.0 ± 0.08	35.7 ± 0.73	47.7 ± 0.25	26.6 ± 1.67
O	36.5 ± 0.09	25.3 ± 0.27	52.3 ± 0.25	30.0 ± 0.82
Cu	-	38.9 ± 0.47	-	43.4 ± 1.04
Ti	0.2 ± 0.01	0.2 ± 0.01	-	-

**Table 4:** The antiviral activity values Mv (log reduction (Vc/Vt) of modified fabrics against VACV, HSV-1, IFV and MHV.

Control Samples	Test Samples	Mv			
		Log Reduction PFU/mL VACV	Log Reduction PFU/mL HSV-1	Log Reduction TCID50/mL IFV	Log Reduction TCID MHV
PET	PET/Cu	0	1	0	1
CO	CO/Cu	2	2	2	1

Where: TCID50—the 50% tissue culture infectious dose; PFU—plaque-forming unit.

**Table 5:** The results of antibacterial activity.

	*Staphylococcus aureus* (ATCC 6538)		*Klebsiella pneumoniae* (ATCC 4352)	
	PET/Cu	CO/Cu	PET/Cu	CO/Cu
Concentration of inoculum, CFU/mL	2.7 × 10^5^		2.8 × 10^5^	
Growth value F—for the control sample (pristine) F = lg C_t_ − lg C_o_	3.73 lg *C_t_*: −8.32 lg C_o_: −4.59	4.95 lg *C*_t_: −9.29 lg *C_o_*: –4.34	4.31 lg *C_t_*: −9.56 lg *C_o_*: −5.25	4.42 lg *C_t_*: −9.61 lg *C_o_*: −5.19
Growth value G—for the test sample (Cu modified) G = lg T_t_ − lg T_o_	−3.10 lg *T_t_:* −1.30 lg *T_o_:* −4.40	0.00 lg *T_t_*: −1.30 lg *T_o_*: –1.30	−2.65 lg *T_t_:* −1.30 lg *T_o_:* −3.95	0.00 lg *T_t_:* −1.30 lg *T_o_:* −1.30
Value of antimicrobial activity A A = (lg *C_t_* − lg *C_o_*) − (lg *T_t_* − lg *C_o_*)	7.02	7.99	8.26	8.31
Time and temperature of incubation	22 h + 48 h (37 ± 2 °C)			

**Table 6:** The values of the contact angle and surface free energy.

Sample		Contact Angle Θ, Deg			Surface Free Energy γ_S_ and Dispersive γ_S_^d^ and Polar Components γ_S_^p^, mJ/m^2^		
		Θ_w_	Θ_F_	Θ_H_	γ_S_	γ_S_^d^	γ_S_^p^
PET	pristine	0.0	18.1 ± 2.1	0.0	72.04	17.21	54.82
	Cu	131.2 ± 1.5	117.1 ± 1.2	0.0	16.43	15.38	1.05
CO	pristine	0.0	22.8 ± 2.2	0.0	71.71	16.81	54.90
	Cu	135.0 ± 1.9	128.6 ± 1.4	0.0	15.26	13.79	1.56

**Table 7:** Results of the Alambeta test.

Sample	λ, Wm^−1^K^−1^	a × 10^−8^, m^2^s^−1^
PET	0.041 ± 0.0008	5.07 ± 0.753
PET/Cu	0.044 ± 0.0012	7.11 ± 1.290
CO	0.038 ± 0.0007	7.90 ± 1.800
CO/Cu	0.039 ± 0.0008	8.10 ± 0.600

## Data Availability

Not applicable.
